# *CD40LG* mutations in Vietnamese patients with X‐linked hyper‐IgM syndrome; catastrophic anti‐phospholipid syndrome as a new complication

**DOI:** 10.1002/mgg3.1732

**Published:** 2021-06-10

**Authors:** Anh Nguyen Lien Phan, Thuy Thi Thanh Pham, Xinh Thi Phan, Nghia Huynh, Tuan Minh Nguyen, Cuc Tran Thu Cao, Duong Thuy Nguyen, Khanh Thi Xuan Luong, Tam Thi Minh Nguyen, Anh Ngoc Kim Tran, Linh Thi Truc Pham, Vy Vuong Thao Nguyen, Sigrid Swagemakers, Chi‐Bao Bui, Petrus Martinus Van Hagen

**Affiliations:** ^1^ Children’s Hospital 1 Ho Chi Minh City Vietnam; ^2^ Functional Genomic Unit DNA Medical Technology Ho Chi Minh City Vietnam; ^3^ Department of Haematology University of Medicine and Pharmacy at Ho Chi Minh City Ho Chi Minh City Vietnam; ^4^ Department of Pathology & Clinical Bioinformatics Erasmus MC Rotterdam The Netherlands; ^5^ School of Medicine Vietnam National University Ho Chi Minh City Vietnam; ^6^ Molecular Genetics City Children’s Hospital Ho Chi Minh City Vietnam; ^7^ Department of Immunology Erasmus MC Rotterdam The Netherlands; ^8^ Department of Internal medicine Division Clinical Immunology Erasmus MC Rotterdam The Netherlands

**Keywords:** anti‐phospholipid syndrome, CD40 ligand, hyper‐IgM syndrome, primary immunodeficiency, whole‐exome sequencing

## Abstract

**Background:**

X‐linked hyper‐IgM syndrome (XHIGM) is a rare primary immunodeficiency caused by CD40 ligand defects.

**Methods:**

We identified three patients with XHIGM in Ho Chi Minh City, Vietnam. Whole‐exome sequencing, immunological analyses and western blot were performed to investigate phenotypic and genotypic features.

**Results:**

Despite showing symptoms typical of XHIGM, including recurrent sinopulmonary infections, oral ulcers and otitis media, the diagnosis was significantly delayed. One patient developed anti‐phospholipid syndrome, which has been documented for the first time in XHIGM syndrome. Two patients had elevated IgM levels and all of them had low IgG levels. Exome sequencing revealed mutations in the *CD40LG* gene: one novel splicing mutation c.156+2T>A and two previously characterised mutations (non‐frameshift deletion c.436_438delTAC, stop‐gain c.654C>A). Due to these mutations, the CD40 ligand was not expressed in any of the three patients, as demonstrated by western blot analysis.

**Conclusion:**

This is the first report of XHIGM syndrome in Vietnam indicates that an effective diagnostic strategy, such as sequencing analysis, contributes to reliable diagnosis and subsequent therapy.

## Graphical abstract

X‐linked hyper‐IgM syndrome (XHIGM) is a rare primary immunodeficiency caused by CD40 ligand defects. Exome sequencing revealed mutations in the CD40LG gene: one novel splicing mutation c.156+2T>A and two previously characterised mutations (non‐frameshift deletion c.436_438delTAC, stop‐gain c.C654A). This first is the first report of XHIGM syndrome in Vietnam indicates that an effective diagnostic strategy, such as sequencing analysis, contributes to reliable diagnosis and subsequent therapy.

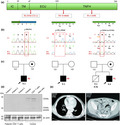

## 1 INTRODUCTION

X‐linked hyper‐IgM syndrome (XHIGM; OMIM: 308230) is the most frequently occurring form of hyper‐IgM syndrome and is associated with mutations in the gene encoding CD40 ligand (CD40L). Patients with XHIGM usually have elevated (or normal) IgM levels and low levels of other immunoglobulin isotypes. Over half of patients have chronic neutropenia that contributes to the development of oral ulcers and opportunistic infections. These patients are prone to developing autoimmune disorders (Notarangelo & Hayward, 2000).

The *CD40LG* (OMIM: 300386) is a member of the tumour necrosis factor (TNF) family, which is expressed by activated T cells. CD40L interacts with CD40 on the surface of B cells to provide essential signals for cell proliferation and immunoglobulin class switching, explaining the defected isotype switch from IgM to IgG, IgA and IgE (Notarangelo & Hayward, 2000; Schönbeck & Libby, 2001).

Anti‐phospholipid syndrome (APS) is an autoimmune disorder characterised by vascular thromboembolism, miscarriages and other pregnancy comorbidities (Radic & Pattanaik, 2018). This autoimmune feature has not been previously reported in patients with XHIGM.

The heterogeneity of XHIGM makes its diagnosis challenging, and accurate and reliable molecular and genetic testing methods are needed to confirm the disorder. There have been several studies on XHIGM (Cabral‐Marques et al., 2014; Wang et al., 2014; Winkelstein et al., 2003); however, no cases have been reported from Vietnam. This work reports the first three Vietnamese patients with XHIGM. Significantly, we characterised one novel mutation in the *CD40LG* gene, which leads to the absence of its protein expression. We also found a novel autoimmune feature (APS) of XHIGM. The results of our study highlight the need for a more effective (genetic) approach to confirm the diagnosis in this part of the world in order to start treating patients in a timely manner.

## 2 MATERIALS AND METHODS

### 2.1 Patients

Patients, from three unrelated and non‐consanguineous families, were recruited from Children's Hospital 1, Ho Chi Minh City (2017–2020) after obtaining informed consent, under the Ethics Review Board of Children's Hospital 1 Ho Chi Minh City. The inclusion criteria were low serum IgG and IgA (two standard deviations below the normal value for their age), normal or elevated serum IgM, and infections that can be expected in XHIGM.

### 2.2 Genetic, immunological and immunoblot analysis

Whole exome library preparation and sequencing were performed using Agilent SureSelect Human All Exon V5 (Agilent Technologies) on a NovaSeq 6000 Sequencing System (Illumina). We applied an in‐house bioinformatics WES analysis pipeline.

The immunoglobulin level was measured using nephelometry. The lymphocyte subsets were analysed by fluorescence‐activated cell sorting using the BD MutitestTM reagents (BD Biosciences).

Polyclonal rabbit anti‐CD40L antibodies (CD40L (N) Antibody; Abiocode) and mouse anti‐β‐actin monoclonal antibodies (Clone C4; Millipore) were used to identify CD40L.

Detailed methods are described in the supporting document and our previous study (Phan et al., 2020).

## 3 RESULTS AND DISCUSSION

### 3.1 Clinical manifestations and laboratory analysis

The onset of clinical manifestation in three patients ranged from 10 days to 11 months. All of them developed signs of immunodeficiency in their first year, agreeing with the 75% cases in the study of Winkelstein et al., (2003). Otitis media and recurrent sinopulmonary infections were observed in all patients, similar to several studies (Gilmour et al., 2003; de la Morena et al., 2017; Wang et al., 2014; Winkelstein et al., 2003). Patient 1 (P1) and patients 2 (P2) presented with *Pneumocystis jirovecii* pneumonia, while P3 had lung abscesses. P1 suffered from *Pseudomonas* *aeruginosa* sepsis. Both P1 and patient (P3) had neutropenia and associated oral ulcers and hypopigmentation. P2 and P3 developed recurrent diarrhoea from 16 months and 10 years, respectively. Protracted or recurrent diarrhoea was a characteristic symptom. Previous reports revealed non‐infectious diarrhoea in more than 50% of patients; yet no etiologic agent was identified in P2 and P3, and P3 later developed Crohn's disease (Wang et al., 2014; Winkelstein et al., 2003). In addition to the infection susceptibility developing before diagnosis, two other clinical events were prominent, that is *P jiroveci* pneumonia and neutropenia. These clinical presentations require prompt consideration for XHIGM diagnosis (Winkelstein et al., 2003). Still, the diagnosis delay hơvaried from 5.5 months (P2) to 30 months (P1) and up to 10 years (P3). Previous studies indicated that positive family history with early death in male members may give aid to the diagnosis (Wang et al., 2014; Winkelstein et al., 2003). In P1, whose brother died from systemic tuberculosis, the diagnosis lag was up to 3 years. Early medical attention did not help diagnose XHIGM due to the lack of appropriate laboratory techniques at that time. All patients had decreased serum IgG levels. While P2 presented with normal IgM and decreased IgA levels, P1 and P3 had elevated IgM, agreeing with 32% of patients in a multi‐centre study (Winkelstein et al., 2003). Lymphocyte (subset) counts were normal, except for P3 who had significantly decreased B cell level and increased double‐negative T cell level (Table 1).

**TABLE 1:** Clinical features, treatments, and genetic findings of Vietnamese patients

Clinical features	Patient 1	Patient 2	Patient 3
Onset‐diagnosis age (m)	6–36	0.5–6	11–130
Diagnosis lag (m)	30	5.5	119
Family history	+	−	−
Otitis media, Recurrent sinopulmonary	+	+	+
Oral ulcer	+	−	+
Neutropenia	+	−	+
Recurrent diarrhoea	−	+	+
IgG	D	D	D
IgM	I	N	I
IgA	N	D	N
B lymphocyte	N	N	D
Pathogen	PJ, SA	PJ	−
Significant events			Lung abscess, Crohn's, APS
Treatments	IVIG	IVIG	IVIG + steroid + warfarin
Genetic findings			
Detected mutation	c.654C>A (exon 5)	c.156+2T>A (intron 1)	c.436_438delTAC (exon 5)
Effect on protein‐Domain	p.Cys218Ter (TNFH)	IVS1+2T>A (ECU)	p.Tyr146del (TNFH)
Novel mutation	−	+	−
CD40 ligand expression	Absent	Absent	Absent
Mother carrier	+	+	−

Abbreviations: APS, anti‐phospholipid syndrome; D, decrease; ECU, extracellular unique domain; I, increase; IVIG, Intravenous immunoglobulin; m, months; minus sign (−), Absent. TNFH, tumour necrosis factor homology domain; N, normal; PJ, *Pneumocystis jiroveci*; positive sign (+), Present; SA, *Staphylococcus aureus*.

**FIGURE 1:**
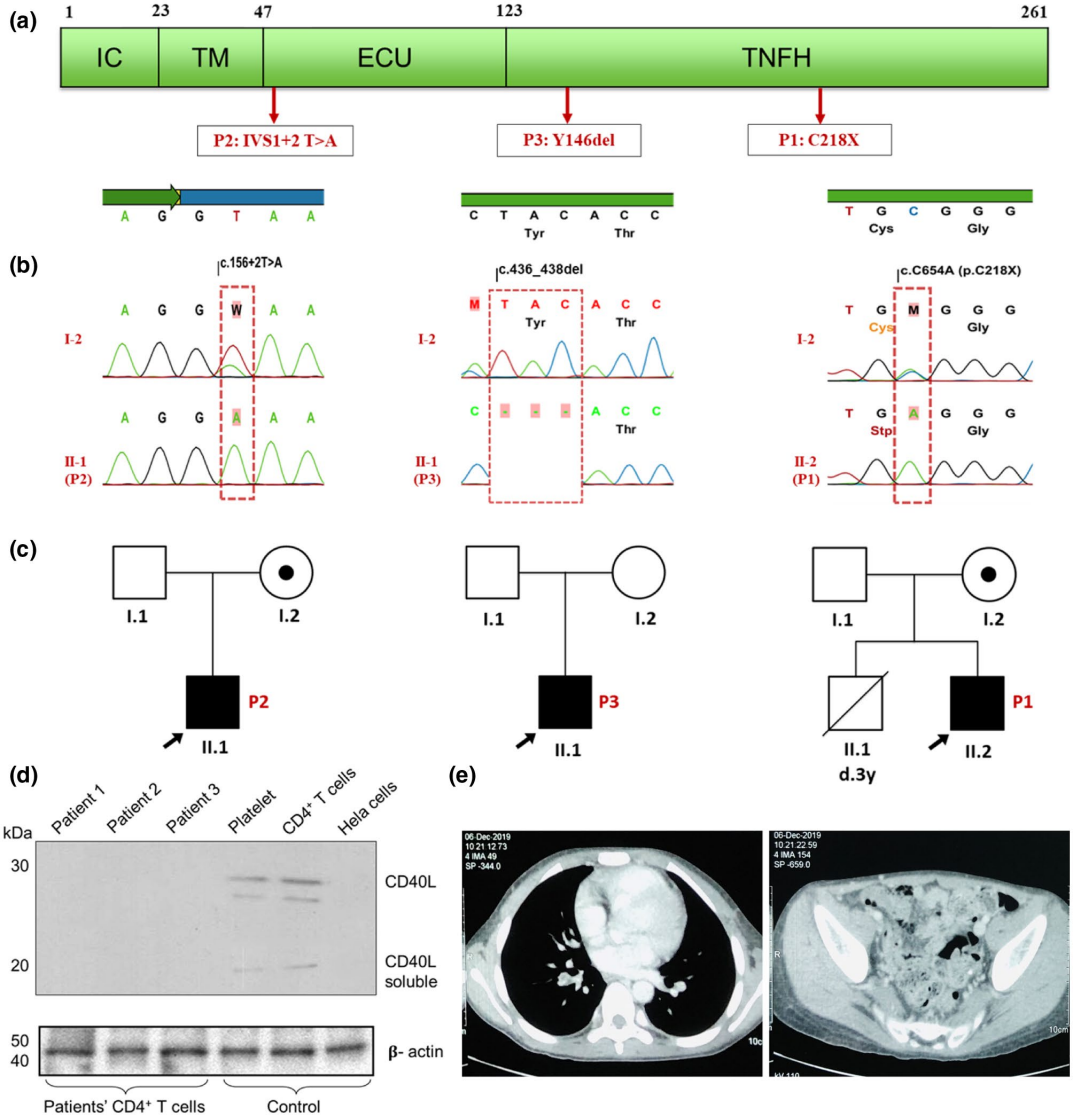
Clinical presentation, genetic analyses, and protein expression of Vietnamese patients. (a) Schematic representation of CD40 ligand domains and identified variants. IC: intracytoplasmic; TM: transmembrane; ECU: extracellular unique; TNFH: tumour necrosis factor homology. (b) Sanger sequencing of *CD40LG* in the probands and their mothers. (c) Pedigree of the three families. Circles: female subjects; squares: male subjects; solid symbols: patients; shaded dot inside a circle: female carrier. The arrows indicate P1, P2, and P3. (d) Western blot analysis reveals the loss of CD40L protein expression in all patients. Soluble CD40L is absent in all patients as compared to the clear bands in the normal control human platelet, CD4+ T cells, and HeLa cells. (e) CT scan shows deep vein thrombosis in the right pulmonary artery and right common femoral vein in P3

Patients with XHIGM may suffer from autoimmune and/or haematologic complications, such as seronegative arthritis, inflammatory bowel disease including Crohn's disease, and haemolytic anaemia (Levy et al., 1997; Qiu et al., 2017; Winkelstein et al., 2003). P3 had Crohn's ileitis and colitis, which were diagnosed by ileocolonoscopy and histopathological examinations. He also developed APS, clinically expressing itself as peripheral oedema dyspnoea, chest pain, and headache. A whole‐body computerised tomography scan confirmed multiple deep vein thrombosis in the superior sagittal sinus, right pulmonary artery, inferior vena cava, common iliac veins and right common femoral vein (Figure 1e). The presence of lupus anticoagulant was determined using the silica clotting time and dilute Russell viper venom time test (Radic & Pattanaik, 2018). The positive results confirmed the presence of (in vitro) inhibitors of haemostasis, supporting the diagnosis of APS. Other anti‐phospholipid tests (anti‐β2 glycoprotein‐I IgG/IgM and anti‐cardiolipin IgG and IgM) were negative (Radic & Pattanaik, 2018). Additional immunological investigations were negative. The APS in P3 was established for the first time in patients with XHIGM. Remarkably, *CD40LG* is listed among the top associated genes for APS (GeneCards database). Moreover, genetic polymorphisms in CD40, the main receptor for CD40L, have been linked to thrombosis in APS patients (Radic & Pattanaik, 2018). These associations suggest that this autoimmune disease might be a feature of XHIGM; nevertheless, appropriate studies need to be performed in an XHIGM cohort to determine the thrombosis frequencies and confirm the positive laboratory tests for lupus anticoagulant, anti‐cardiolipin antibodies and β2‐glycoprotein‐I antibodies.

### 3.2 Mutation analysis and immunoblot analysis

Exome analysis identified *CD40LG* mutations in three patients, all met the classification criteria for pathogenic mutations, following our previous study (Phan et al., 2020). P1 possesses a reported nonsense mutation NM_000074.3: c.654C>A (p.Cys218Ter) (Cabral‐Marques et al., 2014; Lee et al., 2005; Prasad et al., 2005; Wang et al., 2014). P3 has a previously described non‐frameshift deletion NM_000074.3:c.436_438delTAC (p.Tyr146del) (Lee et al., 2005). Both of these mutations on exon 5 affect the tumour necrosis factor homologous (TNFH) domain. A novel splicing mutation, NM_000074.3:c.156+2T>A (IVS1+2T>A), was characterised in P2. Mutations in *CD40LG* are diverse and have different effects on CD40L structure and function, demonstrating its genetic heterogeneity (Notarangelo & Hayward, 2000). 37% of the mutations affect receptor binding or trimerisation (Thusberg & Vihinen, 2007). Mutants clustered at specific positions (Cys218, Thr254, Trp140, IVS1+1, IVS2+1 and IVS4+1) were suggested as mutational hotspots for the *CD40LG* (Lee et al., 2005; Notarangelo et al., 1996). The mutation p.Cys218Ter (P1), might affect disulphide bridge formation (Cabral‐Marques et al., 2014; Prasad et al., 2005; Winkelstein et al., 2003), is usually associated with more severe clinical features (Notarangelo et al., 1996; Wang et al., 2014). The in‐frame deletion at Tyr146 (P3) leads to an amino acid change in the TNFH domain. This and surrounding mutations (p.Tyr145del, p.Thr147Asn) have been reported previously in the HGMD database (retrieved 15/11/2020) and other studies (Lee et al., 2005). Significantly, the novel splicing in intron 1 (P2), c.156+2T>A, is closely located to many characterised mutations (c.154A>T, c.156+1G>T). A mutation at the same position, c.156+2T>C, has been reported (Aghamohammadi et al., 2009). These mutations potentially cause aberrant splicing, resulting in amino acid change in the extracellular unique domain (Table 1, Figure 1a,b).

No significant correlation has been identified between clinical phenotype and *CD40LG* mutation's location (Danielian et al., 2007; Levy et al., 1997; Prasad et al., 2005). Nevertheless, some hypomorphic mutations (p.Thr254Met, p.Arg11Ter) have been documented to associated with “milder” and later‐onset XHIGM (Notarangelo & Hayward, 2000; Prasad et al., 2005). The CD40L residues Y146 and C128 (P1, P3) were shown to make direct contact with the receptor CD40, implying the crucial effect of CD40‐CD40L interaction on these patients’ immune response (An et al., 2011). However, it is unknown if these critical mutations might contribute to the different clinical manifestations in P1 and P3, which were likely more severe than those in P2. More associations should be studied in other families with *CD40LG* mutations to elucidate the phenotypes, prognosis, and therapeutic effects.

Sanger sequencing confirmed the *de novo* mutation in P3 and carrier status in P1’s and P2’s mothers (Figure 1b,c), providing crucial information for female relatives at risk of being XHIGM carriers, suggesting that prenatal testing might be important (Prasad et al., 2005). Thus, identification of *CD40LG* mutations is valued for early diagnosis of XHIGM, appropriate therapeutic approaches, and genetic counselling. Western blot analysis of activated CD4+ T cell extracts showed the absence of CD40L expression and loss of soluble CD40L expression in all patients. This might be the result of a large truncation in CD40L (Table 1, Figure 1c,d).

Noteworthy, we implemented genetic analysis prior to protein expression evaluation, which is not in agreement with the suggested workflow for XHIGM diagnosis at other centres from different regions (França et al., 2019; Gilmour et al., 2003). Nevertheless, given our lab conditions, this approach is more cost‐effective since the analysis of specific proteins (western blot or flow cytometry) is not a regular practice. Moreover, variants detected by WES may indicate other primary immunodeficiencys (PIDs) with a comparable disease spectrum, such as PI3‐kinase delta mutations.

### 3.3 Therapy and course of disease

Treatment of XHIGM syndrome includes IVIG (intravenous immunoglobulin), granulocyte colony‐stimulating factor for neutropenia, prophylactic and therapeutic antibiotics and HSCT (França et al., 2019; de la Morena et al., 2017). Patients who undergo IVIG may still develop infections (Quartier et al., 1999). Our patients were treated regularly with IVIG therapy at 5‐week intervals (P1, P2) and at 4‐week intervals (P3). P3 received IVIG in combination with warfarin and glucocorticosteroid therapy to treat APS and Crohn's disease, respectively. During the 3‐month glucocorticosteroid and 6‐months warfarin therapy, P3 remained free from Crohn's disease‐like manifestations. All of them were free of severe bacterial infections during treatment; however, P1 and P3 still suffered from neutropenia and recurrent oral ulcers. Haematopoietic stem cell transplantation (HSCT) was initiated for P3 due to the severe clinical picture including the initial life‐threatening autoimmune complications. For HSCT, a human leukocyte antigen‐identical match donor is crucial. Because of hepatic diseases, this therapy is not successful in almost 40% of patients. Moreover, only 1/5 of the patients (receiving HSCT or not) reach the age of 25 (de la Morena et al., 2017). Thus, there is an urgent need for more feasible and personalised treatment for XHIGM, such as targeted gene therapy (França et al., 2019; de la Morena et al., 2017).

In summary, we reported for the first time the clinical, immunological, and molecular features of Vietnamese patients with XHIGM. Genetic analysis revealed distinct *CD40LG* mutations, including a novel mutation. We also characterised a unique autoimmune feature: APS, which might be a significant life‐threatening complication of XHIGM that needs special attention. The effectiveness of WES analysis in our setting is a promising approach to diagnose PID in Vietnam.

## ETHICAL COMPLIANCE

The study protocol was approved by the Review Board committee of University of Medicine and Pharmacy at Hochiminh city (No.2020.CH02). The study was conducted in accordance with the Good Clinical Practice and the Declaration of Helsinki. All parents of the child patients provided informed consents prior to study.

## CONFLICT OF INTEREST

The authors declare no conflict of interest.

## AUTHOR CONTRIBUTIONS

Anh. N.L. Phan was the principal clinician in charge of patient care and wrote part of the manuscript. Thuy. T.T. Pham implemented the analysis of WES and wrote part of the manuscript. P.M. van Hagen and Chi‐Bao. Bui were co‐principal investigators on this study. Linh. T.T. Pham and Vy. V.T. Nguyen conducted the WES analysis and Sanger sequencing. S. Swagemakers performed bio‐informatics and databank analysis. Xinh Phan and Nghia Huynh performed the immunological analysis. Tuan. M. Nguyen, Cuc. T.T. Cao, Duong. T. Nguyen, Tam. T.M. Nguyen, Khanh. T.X. Luong, and Anh. N.K. Tran were involved in the management of the patients. All authors reviewed the manuscript and contributed to the final manuscript.

## Supporting information

Supplementary Material

Supplementary Material

## Data Availability

The data that support the findings of this study are not publicly available due to privacy. However, the data are available on request from the corresponding author.
