# Supplementary material for: CD40LG mutations in Vietnamese patients with X‐linked hyper‐IgM syndrome; catastrophic anti‐phospholipid syndrome as a new complication
Source: Mol Genet Genomic Med. 2021 Jun 10;9(8):e1732. doi: 10.1002/mgg3.1732 (PMC8404229; doi:10.1002/mgg3.1732)
Supplement: Supplementary file 1 — Supplementary Material [file MGG3-9-e1732-s001.docx]

# SUPPORTING INFORMATION

**CD40LG MUTATIONS IN VIETNAMESE PATIENTS WITH X-LINKED HYPER-IgM SYNDROME; CATASTROPHIC ANTI-PHOSPHOLIPID SYNDROME AS A NEW COMPLICATION**

***Supporting table listing the variants found in 3 patients after the variant priorization steps (pathogenicity, gene panel, segregation, frequency)***

(*) WES was implemented following the 2015 American College of Medical Genetics and Genomics/Association for Molecular Pathology (ACMG/AMP) guidelines. We applied the in-house bioinformatics pipeline for WES analysis, as described in our previous study. The variations with minor allele frequencies <0.05 in any of these databases: dbSNP, 1000 Genomes Project, ExAC and NHLBI Exome Sequencing Project (ESP6500) were filtered out. The analysis performed include computational analysis of DANN, Genomic Evolutionary Rate Profiling++, Likelihood Ratio Test, MutationTaster, Functional Analysis through Hidden Markov Models‐MKL coding, Combined annotation-dependent depletion, and EIGEN score. The variations with minor allele frequencies <0.05 in any of these databases: dbSNP, 1000 Genomes Project, The Genome Aggregation Database (gnomAD) and NHLBI Exome Sequencing Project (ESP6500) were filtered out. The genetic primary immunodeficiency panel (IUIS2017) was applied to identify variants in the PID genes.

| **PATIENT 1** | | | | | | | | | | | | |
| --- | --- | --- | --- | --- | --- | --- | --- | --- | --- | --- | --- | --- |
| **CHR** | **POS** | **REF** | **ALT** | **Gene** | **DiseaseID** | **Inheritance** | **Zygosity** | **Function** | **AAChange** | **gnomAD_genome_ALL** | **esp6500siv2_all** | **1000g2015aug_all** |
| chr1 | 198608242 | C | T | PTPRC | Severe combined immunodeficiency, autosomal recessive, T-cell negative, B-cell positive, NK cell positive | AR | HET | . | NM_002838:c.-157C>T;NM_080921:c.-157C>T;NM_001267798:c.-157C>T | 0.0078 | . | 0.0219649 |
| chr1 | 198697438 | G | A | PTPRC | Severe combined immunodeficiency, autosomal recessive, T-cell negative, B-cell positive, NK cell positive | AR | HET | . | . | 0.0009 | 0.0015 | 0.0007987 |
| chr3 | 52413894 | C | T | DNAH1 | Ciliary dyskinesia, primary, 37 | AR | HET | . | . | . | . | . |
| chr5 | 77523152 | T | C | AP3B1 | Hermansky-Pudlak syndrome 2 | AR | HET | . | . | . | . | . |
| chr7 | 21757560 | G | A | DNAH11 | Ciliary dyskinesia, primary, 7 | AR | HET | . | . | 0.0003 | . | 0.0003994 |
| chr16 | 67692871 | G | T | ACD | Dyskeratosis congenita 6; Dyskeratosis congenita 7 | AD/AR | HET | nonsynonymous SNV | ACD:NM_001082486:exon7:c.C863A:p.P288H,ACD:NM_001082487:exon7:c.C854A:p.P285H,ACD:NM_022914:exon7:c.C854A:p.P285H | . | . | . |
| chr17 | 43348646 | C | G | MAP3K14 | Primary immunodeficiency with multifaceted aberrant lymphoid immunity | AR | HET | . | . | 0.0038 | . | 0.0023962 |
| chr17 | 43364087 | G | T | MAP3K14 | Primary immunodeficiency with multifaceted aberrant lymphoid immunity | AR | HET | unknown | UNKNOWN | . | . | . |
| chrX | 135741442 | C | A | CD40LG | Immunodeficiency, with hyper-IgM, type 1 | XL | HEM | stopgain | CD40LG:NM_000074:exon5:c.C654A:p.C218X | . | . | . |
|  |  |  |  |  |  |  |  |  |  |  |  |  |
|  |  |  |  |  |  |  |  |  |  |  |  |  |

| **PATIENT 2** | | | | | | | | | | | | |
| --- | --- | --- | --- | --- | --- | --- | --- | --- | --- | --- | --- | --- |
| **CHR** | **POS** | **REF** | **ALT** | **Gene** | **DiseaseID** | **Inheritance** | **Zygosity** | **Function** | **AAChange** | **gnomAD_genome_ALL** | **esp6500siv2_all** | **1000g2015aug_all** |
| chr1 | 11103581 | C | T | MASP2 | MASP2 deficiency | AR | HET | nonsynonymous SNV | MASP2:NM_006610:exon5:c.G556A:p.G186S | 3.2E-05 | . | . |
| chr2 | 32475600 | A | C | NLRC4 | Autoinflammation with infantile enterocolitis (AIFEC); Familial cold autoinflammatory syndrome 4 | AD | HET | nonsynonymous SNV | NLRC4:NM_001199138:exon4:c.T1333G:p.S445A,NLRC4:NM_001199139:exon4:c.T1333G:p.S445A,NLRC4:NM_021209:exon4:c.T1333G:p.S445A | . | . | . |
| chr2 | 32475770 | C | T | NLRC4 | Autoinflammation with infantile enterocolitis (AIFEC); Familial cold autoinflammatory syndrome 4 | AD | HET | nonsynonymous SNV | NLRC4:NM_001199138:exon4:c.G1163A:p.R388Q,NLRC4:NM_001199139:exon4:c.G1163A:p.R388Q,NLRC4:NM_021209:exon4:c.G1163A:p.R388Q | 3.2E-05 | . | 0.0001997 |
| chr3 | 52428491 | G | A | DNAH1 | Ciliary dyskinesia, primary, 37 | AR | HET | nonsynonymous SNV | DNAH1:NM_015512:exon67:c.G10637A:p.R3546Q | 9.7E-05 | . | 0.000599 |
| chr4 | 126336542 | G | C | FAT4 | Hennekam lymphangiectasia-lymphedema syndrome 2 | AR | HET | nonsynonymous SNV | FAT4:NM_001291285:exon5:c.G6424C:p.V2142L,FAT4:NM_001291303:exon5:c.G6424C:p.V2142L,FAT4:NM_024582:exon5:c.G6424C:p.V2142L | . | . | . |
| chr7 | 21901544 | T | A | DNAH11 | Ciliary dyskinesia, primary, 7 | AR | HET | nonsynonymous SNV | DNAH11:NM_001277115:exon69:c.T11276A:p.I3759N | . | . | . |
| chr10 | 6066218 | G | A | IL2RA | Immunodeficiency 41 with lymphoproliferation and autoimmunity | AR | HET | nonsynonymous SNV | IL2RA:NM_000417:exon3:c.C356T:p.A119V,IL2RA:NM_001308242:exon3:c.C356T:p.A119V,IL2RA:NM_001308243:exon3:c.C356T:p.A119V | . | 0.000077 | . |
| chr19 | 863220 | C | G | CFD | Complement factor D deficiency | AR | HET | nonsynonymous SNV | CFD:NM_001317335:exon5:c.C765G:p.I255M,CFD:NM_001928:exon5:c.C744G:p.I248M | 0.0077 | 0.0005 | 0.0391374 |
| chr2 | 202052464 | CAG | C | CASP10 | Autoimmune lymphoproliferative syndrome, type IIA | AD | HET | frameshift deletion | CASP10:NM_001206524:exon3:c.384_385del:p.N130Lfs*15,CASP10:NM_001206542:exon3:c.384_385del:p.N130Lfs*15,CASP10:NM_001230:exon3:c.384_385del:p.N130Lfs*15 | . | . | . |
| chr12 | 2788638 | T | C | CACNA1C | Brugada syndrome 3; Timothy syndrome | AD | HET | nonsynonymous SNV | CACNA1C:NM_001129837:exon41:c.T5144C:p.V1715A,CACNA1C:NM_001129838:exon41:c.T5144C:p.V1715A | 9.7E-05 | . | 0.0009984 |
| chrX | 135730565 | T | A | CD40LG | Immunodeficiency, with hyper-IgM, type 1 | XL | HEM | splicing | NM_000074:exon1:c.156+2T>A | . | . | . |

| **PATIENT 3** | | | | | | | | | | | | |
| --- | --- | --- | --- | --- | --- | --- | --- | --- | --- | --- | --- | --- |
| **CHR** | **POS** | **REF** | **ALT** | **Gene** | **DiseaseID** | **Inheritance** | **Zygosity** | **Function** | **AAChange** | **gnomAD_genome_ALL** | **esp6500siv2_all** | **1000g2015aug_all** |
| chr1 | 167487808 | TCCC | T | CD247 | Immunodeficiency due to defect in CD3-Zeta | AR | HET | . | NM_000734:c.-107_-109delGGG;NM_198053:c.-107_-109delGGG | 0.0026 | . | 0.0105831 |
| chr2 | 204822723 | T | C | ICOS | Immunodeficiency, common variable, 1 | AR | HET | . | . | 0.0099 | . | 0.0369409 |
| chr2 | 217329432 | CA | C | SMARCAL1 | Schimke immunoosseous dysplasia | AR | HET | . | . | 0.0074 | . | . |
| chr3 | 195801917 | CA | C | TFRC | Immunodeficiency 46 | AR | HET | . | . | 0.0077 | . | . |
| chr5 | 169116406 | G | A | DOCK2 | Immunodeficiency 40 | AR | HET | . | . | 0.0009 | . | 0.0017971 |
| chr5 | 169267720 | G | T | DOCK2 | Immunodeficiency 40 | AR | HET | . | . | 0.0088 | 0.0054 | 0.0077875 |
| chr9 | 139264900 | C | A | CARD9 | Candidiasis, familial, 2 | AR | HET | . | . | 0.0005 | . | 0.0031949 |
| chr11 | 118210018 | G | A | CD3D | Immunodeficiency 19 | AR | HET | . | . | 0.0009 | . | 0.004393 |
| chr19 | 2129221 | CA | C | AP3D1 | Hermansky-Pudlak syndrome 10 | AR | HET | . | . | 0.0044 | 0.0058 | 0.0035943 |
| chr19 | 6709645 | A | C | C3 | Complement component 3 deficiency; Hemolytic uremic syndrome, atypical, susceptibility to, 5 | AR | HET | . | . | 0.0018 | . | . |
| chr19 | 18174946 | TTC | T | IL12RB1 | Immunodeficiency 30 | AR | HET | . | . | 0.0022 | . | . |
| chr20 | 397940 | C | T | RBCK1 | Polyglucosan body myopathy 1 | AR | HET | . | . | 0.0053 | 0.0003 | 0.0173722 |
| chrX | 135741220 | ATAC | A | CD40LG | Immunodeficiency, with hyper-IgM, type 1 | XL | HOM | nonframeshift deletion | CD40LG:NM_000074:exon5:c.433_435del:p.Y146del | . | . | . |

***(**) Column header and meaning***

| ***Abbreviation*** | ***Meaning*** |
| --- | --- |
| CHR | Chromosome |
| POS | Position |
| REF | Reference allele |
| ALT | Alternative allele |
| Gene | Gene name |
| DiseaseID | Disease name |
| Zygosity | HET: heterozygous, HOM: homozygous, HEM: hemizygous |
| AAChange | Change in amino acid |
| gnomAD_genome_ALL | Minor allele frequency on The Genome Aggregation Database (gnomAD) |
| esp6500siv2_all | Minor allele frequency in the NHLBI Exome Sequencing Project (ESP6500) |
| 1000g2015aug_all | Minor allele frequency in the 1000 Genomes Project |
