# Supplementary material for: CD40LG mutations in Vietnamese patients with X‐linked hyper‐IgM syndrome; catastrophic anti‐phospholipid syndrome as a new complication
Source: Mol Genet Genomic Med. 2021 Jun 10;9(8):e1732. doi: 10.1002/mgg3.1732 (PMC8404229; doi:10.1002/mgg3.1732)
Supplement: Supplementary file 2 — Supplementary Material [file MGG3-9-e1732-s002.docx]

**SUPPORTING INFORMATION**

**CD40LG MUTATIONS IN VIETNAMESE PATIENTS WITH X-LINKED HYPER-IgM SYNDROME; CATASTROPHIC ANTI-PHOSPHOLIPID SYNDROME AS A NEW COMPLICATION**

**MATERIAL AND METHOD**

## Immunological analysis

The serum immunoglobulin level was measured using nephelometry. The lymphocyte subsets from peripheral blood samples were analysed by fluorescence-activated cell sorting using the BD MutitestTM reagents (BD Multitest IMK kit and BD Multitest TM 6 color TBNK; BD Biosciences, San Jose, CA, USA), following the manufacturer’s guidelines.

## Genetic analysis

Genomic DNA was extracted from peripheral blood using the QIAamp DNA Blood Mini Kit (#51104; QIAGEN, Netherlands). This was followed by whole exome library preparation and sequencing, which was performed by Macrogen (South Korea), using Agilent SureSelect Human All Exon V5 (Agilent Technologies, Santa Clara, California, USA) on a NovaSeq 6000 Sequencing System (Illumina, Seoul, South Korea). Paired FASTQ reads were processed by FASTQC to obtain useful diagnostics such as Phred-score distribution along the reads, GC content distribution, read-length distribution, sequence duplication level. Trimmomatic was subsequently used for removing contaminated sequencing adapters; removing leading and trailing low quality or N bases below quality 3; scanning the read with a 4-base wide sliding window, cutting when the average quality per base drops below 15; and dropping reads below a length of 36. Pre-processed read pairs were then mapped to hg19 reference genome (from UCSC) by BWA-mem. Additional processing included: MarkDuplicates by PICARD, BaseQualityScoreRecalibration by GATK. VCF files were generated with GATK HaplotypeCaller; filtrated by GATK VariantFiltration, SNP (QD < 2.0, FS > 60.0, MQ < 40.0, MQRankSum < -12.5, ReadPosRankSum < -8.0) and INDEL (QD < 2.0, FS > 200.0, ReadPosRankSum < -20.0), respectively. Finally, ANNOVAR was used to intersect variant annotations from UCSC RefSeq, dbSNP 150, gnomAD, ESP6500, ExAC, 1000G, dbNSFPv3.5.

WES was implemented following the 2015 American College of Medical Genetics and Genomics/Association for Molecular Pathology (ACMG/AMP) guidelines.^1^ We applied the in-house bioinformatics pipeline for WES analysis, as described in our previous study.^2^ The variations with minor allele frequencies <0.05 in any of these databases: dbSNP, 1000 Genomes Project, The Genome Aggregation Database (gnomAD) and NHLBI Exome Sequencing Project (ESP6500) were filtered out. The analysis performed include computational analysis of DANN, Genomic Evolutionary Rate Profiling++, Likelihood Ratio Test, MutationTaster, Functional Analysis through Hidden Markov Models‐MKL coding, Combined annotation-dependent depletion, and EIGEN score. The genetic primary immunodeficiency (PID) panel (2017) was applied to identify variants in the PID genes.^3^ The detected mutations were confirmed by Sanger sequencing.

## Immunoblot analysis

The western blot captures the CD40L protein expression by using an anti-CD40 polyclonal antibody directed against the amino terminus of CD40. Like other TNF family ligands, CD40L is naturally expressed as a membrane-bound molecule, and it gets cleaved from the membrane into its soluble form upon binding with the CD40 receptor. Protein lysates derived from the peripheral blood mononuclear cells, platelets and Hela cells of the patients and healthy controls, platelets and Hela cells were resolved via sodium dodecyl sulphate polyacrylamide gel electrophoresis and then transferred to nitrocellulose membranes. Polyclonal rabbit anti-CD40L antibodies (CD40L (N) Antibody; Abiocode, CA, United States) and mouse anti-β-actin monoclonal antibodies (Clone C4; Millipore, Germany) were used to identify CD40L. Normalization was performed with β-actin as a loading control.

**REFERENCES**

1.         Richards S, Aziz N, Bale S, et al. Standards and guidelines for the interpretation of sequence variants: A joint consensus recommendation of the American College of Medical Genetics and Genomics and the Association for Molecular Pathology. *Genetics in Medicine*. 2015;17(5):405-424. doi:10.1038/gim.2015.30

2.         Phan ANL, Pham TTT, Huynh N, et al. Novel compound heterozygous stop-gain mutations of LRBA in a Vietnamese patient with Common Variable Immune Deficiency. *Molecular Genetics & Genomic Medicine*. 2020;8(5):e1216. doi:https://doi.org/10.1002/mgg3.1216

3.         Picard C, Bobby Gaspar H, Al-Herz W, et al. International Union of Immunological Societies: 2017 Primary Immunodeficiency Diseases Committee Report on Inborn Errors of Immunity. *Journal of Clinical Immunology*. 2018;38(1):96-128. doi:10.1007/s10875-017-0464-9
